# Defending and including students with special educational needs: a qualitative study with Chilean students

**DOI:** 10.3389/fpsyg.2026.1846202

**Published:** 2026-06-08

**Authors:** David Cuadra-Martínez, Millaray Monárdez-Monárdez, José Espinosa-Caicedo, Daniel Pérez-Zapata, José Sandoval-Díaz, Rodrigo Landabur, Pablo Castro-Carrasco, Aldo Vera-Calzaretta

**Affiliations:** 1 Departamento de Psicología, Universidad de Atacama, Copiapó, Atacama, Chile; 2 School of Psychology, University of Birmingham, Birmingham, United Kingdom; 3 Centro de Estudios del Ñuble, Universidad del Biobío, Chillán, Chile; 4 Departamento de Psicología, Universidad de La Serena, La Serena, Chile; 5 Centro de Investigación en Teoría Subjetivas, Departamento de Psicologia, Universidad de La Serena, La Serena, Chile; 6 Departamento de Kinesiología, Universidad de Atacama, Copiapó, Atacama, Chile

**Keywords:** defending, including, prosocial behavior, special educational needs, students, subjectivity

## Abstract

**Introduction:**

This study describes and interprets the subjective theories that a group of students from a public and state secondary school in the Atacama Region, Chile, hold regarding high-cost prosocial behavior on students with special educational needs (SEN).

**Methods:**

A qualitative methodology was employed, with an instrumental case study design. A total of 11 students, selected through theoretical sampling, participated in the study. Episodic interviews and a focus group were conducted, with the data being analyzed using thematic coding, grounded theory procedures, and specific analysis of subjective theories.

**Results:**

Findings show that high-cost prosocial behavior is linked to values of justice and inclusion, entails social risks for the benefactor, and has positive effects on well-being, educational inclusion, the academic performance of students with special educational needs, and school climate.

**Discussion:**

Family, school, and group factors are discussed in connection with the promotion of this type of prosociality, highlighting its formative potential with a view to pursuing a fairer, more inclusive educational environment.

## 1 Introduction

Despite the worldwide progress made in the educational inclusion sphere, students with disabilities remain as one of the most excluded groups in schools (UNESCO, 2025a). In Latin America, 19 million of students with special educational needs (SEN) face barriers to education access, have lower levels of social involvement, and stay in educational systems for shorter periods than their non-SEN peers (UNESCO, 2025b). Among them, those who have trouble communicating and taking care of themselves are at a greater risk of dropping out the educational system (UNESCO, 2024).

Students with SEN require specific educational support due to conditions associated with disabilities, learning difficulties, behavioral disorders, or socio-educational vulnerability (Carmona and Montanero, 2025). In 2025, 13% of students attending Chilean schools were enrolled in school integration projects (i.e., a system-wide inclusive strategy through which special educational support is provided to students with SEN). Within this subset, 28% had permanent SEN (students who require special support throughout their school education), while the remaining 72% had been diagnosed with temporary SEN (students who require specific, temporary support; Faúndez et al., 2025).

To achieve social inclusion, SEN students require support from their schools. Findings show that they have problems integrating socially, participating in school life, and developing positive interpersonal relationships with their peers at school, which might result in greater learning difficulties and less motivation to learn (Arroyo and Toro-Mayorga, 2021). In addition, students with SEN have considerably higher rates of social rejection, emotional distress, and exclusion risk (Carmona and Montanero, 2025). In countries such as Sweden, Finland, and the United States, researchers have found that these students are twice as likely to be victims of school violence compared to their peers without SEN (Álvarez-Guerrero et al., 2023).

In Chilean school settings, SEN students are taught in regular classrooms. This can take the form of total inclusion—when students with and without SEN learn together in the same classroom—or partial inclusion—when they share only certain learning activities (Holzer and Moser, 2025). Evidence shows that inclusive education is more difficult to implement successfully in secondary schools than in primary schools, as the secondary curriculum places greater emphasis on content acquisition and the structure of classes requires higher levels of student autonomy (Holzer and Moser, 2025). In addition, the proportion of SEN students who complete secondary education is lower compared with the general population (Dell'Anna et al., 2025).

In line with the reasons presented above, student participation is essential for achieving inclusive school environments and a positive school climate (Ullah and Samad, 2025). Encouraging students to interact positively with their SEN peers has shown to improve communication skills, reciprocal support, and social acceptance (Lin et al., 2025). In this context, students' prosocial behavior may be fundamental for fostering educational settings that support the inclusion and learning of students with SEN.

Prosocial behavior is a multidimensional construct that encompasses actions performed in order to benefit one or more people and that, in general, has positive consequences for society, as it involves helping others through behavior such as supporting, sharing, empathizing, and comforting (Padilla-Walker et al., 2018; Septarini et al., 2025). In schools, prosocial behavior has been linked to positive school climates (Wang et al., 2022), reduced violence, and higher rates of inclusion and well-being (Li et al., 2023).

Some types of prosocial behavior entail major “sacrifices” and risks for the benefactor (Pfattheicher et al., 2022), standing apart from other types of prosocial behavior because of its high cost. This behavior type is known as high-cost prosocial behavior (HCPB, Eisenberg and Spinrad, 2014; Padilla-Walker et al., 2018). HCPB is defined as an infrequent behavior associated with a higher degree of risk and cost for the person who performs it, as it involves defending individuals who may be perceived as “victims” (Nielson et al., 2017) and including those who are excluded (Bergin et al., 2003). This type of behavior requires a substantial investment of cognitive, emotional, and behavioral resources from the benefactor (Nolen, 2016; Padilla-Walker et al., 2018).

(Bergin et al. 2003) found that adolescents who engage in defensive HCPB at school exhibit higher emotional regulation, demonstrated by their willingness to protect peers who are being attacked. In contrast, students who engage in inclusive behavior show friendly actions toward peers who have been excluded from school activities.

Findings show that secondary students are more likely to engage in defensive HCPB for the benefit of their peers compared with students in other educational stages (Lindstrom et al., 2013). However, there is less evidence on how this behavior develops over the course of adolescence. For example, (Waasdorp et al. 2022) note that defensive HCPB is more prevalent at the start of secondary education. In contrast, (Hammond and Brownell 2015) point out that prosocial behavior is less common in early adolescence and tends to increase with age due to a greater need for social integration.

Regarding sex differences in younger populations, researchers have found that girls tend to exhibit more prosocial behavior aimed at comforting others, whereas boys are more likely to engage in riskier forms of prosocial behavior, such as intervening physically to stop aggression (Hammond and Brownell, 2015). However, (Nielson et al. 2017) suggest that the disparities observed between boys' and girls' levels of prosociality may stem from different ways of behaving prosocially as a result of gender stereotypes—an effect that becomes more pronounced during adolescence.

From a psychosocial perspective, HCPB is influenced by a range of psychological, social, and contextual factors. At the psychological level, HCPB has been linked to stronger moral development and prosocial identity (Gini et al., 2011), moral reasoning (Carlo et al., 2010), empathy (Paciello et al., 2013), and self-efficacy (Tomar, 2023). At the social and contextual level, the norms and values of social groups play a substantially important role. In educational settings, students' interactions are fundamental for the development of this behavior. Research shows that starting school gives students the opportunity to recognize their peers‘ emotions and become aware of the school's expectations, which is likely to contribute to the development of prosociality toward peers (Hammond and Brownell, 2015). Increased reasoning capabilities also enable them to understand that, on some occasions, it is necessary to do wrong to attain a greater good (e.g. breaking school norms to defend a peer, Hammond and Brownell, 2015). (Evans and Smokowski 2015) found that student prosociality, via students who witness school violence, is linked to the support friends and teacher staff. Thus, interacting with unknown students at school might establish bonds of friendship and equip them to learn and engage in HCPB in educational settings (Padilla-Walker et al., 2018).

Defending and including others are forms of HCPB because they involve effort, sacrifice, and even personal risk for the benefactor (Hammond et al., 2023; Padilla-Walker et al., 2018). Evidence from a study with secondary students in Tanzania shows that sighted adolescents often helped their visually impaired peers with social and academic challenges, particularly when friendships were present and the school fostered an inclusive culture (Milinga and Possi, 2015). The authors also found that empathic responses—such as reduced distress when understanding the difficulties faced by peers with disabilities—further encouraged prosocial behavior in these settings.

Similarly, a systematic review by (Woodgate et al. 2019) found that prosocial behavior among secondary students toward peers with disabilities fosters a stronger sense of belonging and acceptance, reduces prejudices toward disability, and enhances empathy. However, the authors also noted that paternalistic forms of support can create dependency among beneficiaries and impose significant costs on benefactors, whose social status may be lowered if peers with negative views of SEN reject them.

Despite the above, a study by (Siperstein et al. 2022) on inclusive behavior in school contexts found that students perceive inclusive behavior as requiring little effort and primarily involving a positive attitude toward educational inclusion, with respect and cordiality toward peers with SEN forming the basis of their interactions.

As students with SEN are particularly vulnerable to violence and social exclusion (Carmona and Montanero, 2025; Cerda-Etchepare et al., 2022), it is essential to understand how and why their peers defend and include them. Research on inclusive education is currently seen as a priority, as more empirical evidence is needed to understand the adaptation and educational progress of students with SEN (Dell'Anna et al., 2025), particularly regarding their inclusion and protection from violence (UNICEF, 2025). Moreover, addressing this issue through students' experiences and subjective perspectives is essential, given the central role that current educational policies assign to them in promoting a positive school climate (Arfah et al., 2025).

The present study draws on students' experiences to understand HCPB toward peers with SEN. To this end, we examined the subjective theories held by a group of Chilean secondary school students regarding this behavior. Subjective theories are a form of personal knowledge characterized by an argumentative or explanatory structure (Flick, 2018). Through these intuitive explanations, individuals make sense of how the world works and of their own actions (Catalán, 2018). This type of subjectivity also guides decision-making and shapes behavior to some extent (Flick, 2018).

Although previous research has shown that subjective interpretations shape prosocial behavior—for example, studies with University students have demonstrated that benefactors' personal explanations influence both their motivation to help and the conditions under which helping is inhibited (Alvarado et al., 2019)—no empirical work has examined this relationship in secondary school populations or in the specific context of high-cost prosocial behavior toward peers with SEN. This absence of evidence underscores the need to explore how adolescents understand and explain their own helping actions.

In this context, our study addresses the following research question: What subjective theories do students from a public secondary school in the Atacama Region of Chile hold regarding HCPB toward peers with special educational needs (SEN)? The general objective is to describe and interpret these subjective theories. The specific aims are: (a) to describe the subjective meaning students attribute to HCPB directed toward peers with SEN; (b) to identify the psychosocial factors that, according to students' subjective theories, promote or inhibit this behavior; (c) to examine the subjective theories that explain the perceived impact of HCPB on students with SEN, on school climate, and on the benefactor; and (d) to propose a comprehensive model of HCPB and SEN informed by the subjective theories identified.

## 2 Method

### 2.1 Type of study, methodology, and design

Given that the phenomenon was researched from the perspective of the participants' subjective knowledge, which demanded a flexible approach, the study is descriptive-interpretative in nature and utilizes a qualitative methodology (Flick, 2018). Based on an instrumental case study design (Stake and Visse, 2026), interviews were conducted with a group of secondary school students attending a public secondary school in Atacama, Chile, with an 80% social vulnerability rate, indicating that the majority of the student body is socioeconomically at-risk.

### 2.2 Sampling and participants

Theoretical sampling was used (Glaser and Strauss, 2006), starting with 5 students of both sexes from a public secondary school located in the Atacama Region, Chile, who represented a range of educational levels. Qualitative analysis of the first interviews oriented the information collection process, leading to the incorporation of students who self-reported experiences of inclusion and defense of peers with SEN, along with family and friendship ties with people with this condition. Theoretical saturation was achieved with a 10-student sample, as successive interviews no longer generated substantially new themes, sub-themes, or subjective theories relevant to the study objectives. In addition, the relationships among categories became recurrent and sufficiently developed to support the interpretative model. Finally, in order to strengthen the credibility of the results, they were communicatively validated through an 8-student discussion group, 7 of whose members had already been interviewed. In the discussion group, the preliminary interpretations of the study were shared and discussed with key participants in order to contrast these findings with their experiences and analytically adjust the emerging categories (Flick, 2018).

To be included in the study, potential participants had to be enrolled in the school. Students whose health prevented them from completing the interview on the day it was administered were excluded (Table 1).

**Table 1:** Characteristics of the sample.

Study participants	Grade				
	9th	10th	11th	12th	Total
Man	1	0	1	1	3
Woman	4	1	2	1	8
Students who self-report HCPB toward peers with SEN	2	1	2	1	6
Students reporting classmates with SEN	3	0	1	0	4
Students reporting family members or friends with SEN	2	0	1	1	4

Students in Chile must complete 4 years of secondary education.

### 2.3 Information collection procedure

This study was conducted as part of Fondecyt Regular Project no. 1250553, which was approved by the research ethics board of a University in Northern Chile (Certificado N° 04/26). The participating students were contacted through the administrators of the host school and through their parents. The goals and scientific-ethical criteria of the study were explained to the students and their parents, who were required to sign informed consent and assent documents.

#### 2.3.1 Episodic interview

Ten episodic interviews were conducted. According to (Flick 2018), these interviews are ideally suited to the purposes of the study, since they utilize experiential knowledge to access subjective theories, facilitating the interviewees‘ production of subjective explanations. In this study, the participants were permanently asked to report experiences connected with the topic studied, which enabled them to produce explanations of HCPB toward peers with SEN based on the episode narrated. The thematic script covered the following issues: (a) the meaning of HCPB and that of HCPB toward peers with SEN, (b) the factors that inhibit or foster HCPB toward peers with SEN, and (c) the impact of HCPB on students with SEN, on school climate, and on the benefactor. The interviews were conducted in person, in the school building, and during regular school hours. They lasted between 30 and 45 min each and were audio recorded with the students' authorization.

#### 2.3.2 Discussion group

To validate the preliminary results stemming from the interviews, an 8-student discussion group was set up, 7 of whose members had taken part in the episodic interviews. The discussion group, which lasted approximately 1 h and was audio recorded, was an opportunity to share the preliminary results and explore the degree to which they matched the students' subjective theories.

### 2.4 Data analysis procedure

Content analysis was performed, combining thematic coding (Flick, 2018), some procedures of Grounded Theory (Glaser and Strauss, 2006), and specific analysis of subjective theories (Catalán, 2018). These approaches were combined into a coherent analytical strategy in which each method contributed to different levels of interpretation: thematic coding supported descriptive analysis, Grounded Theory procedures enabled the construction of relationships between categories, and the analysis of subjective theories allowed the identification of underlying explanatory structures.

The coding process was carried out by three researchers. Initially, the interview and discussion group transcripts were analyzed independently. Subsequently, regular team meetings were held to compare coding decisions, discuss divergent interpretations, and refine categories through consensus. This process constituted investigator triangulation and was complemented by methodological triangulation through the use of two data collection techniques: episodic interviews and a discussion group used for communicative validation of the preliminary findings. Together, these procedures strengthened the credibility and consistency of the analysis.

To address the descriptive objectives of the study (objectives a, b, and c) thematic coding was performed. In thematic coding, qualitative data analysis is conducted considering each information unit as a single case. In this study, each interview and discussion group was considered as one case. The first step consisted in an intra-case analysis, which comprised (a) a brief description of the case, (b) the generation of a motto –a statement typical of the case that conveys its association with the topic researched–, and (c) the identification of themes and sub-themes represented by subjective theories, guided by the objectives of the study). In the second phase, an inter-case analysis was performed, which consisted in identifying the themes and sub-themes common to all cases in order to produce a common thematic structure (Flick, 2018).

To address the interpretative objective of the study (objective d), axial and selective coding steps were performed (Glaser and Strauss, 2006). In axial coding, connections were identified between the sub-themes of each thematic category described, whereas in selective coding, associations were established between all the thematic categories to propose an interpretative explanatory model. These results were presented using a diagram and a brief written explanation (Flick, 2018).

To identify the subjective theories present in the text, the proposal advanced by (Catalán 2018) was adopted. Subjective theories were identified through explicit argumentative formulations (e.g., “if... then...”, “this happened because...”) or, when causal links were implicit, through the cautious reconstruction of explanatory relations embedded in participants' narratives (e.g., sequences linking emotions, motives, and actions). These interpretative inferences were grounded in the textual data, discussed among three researchers, and contrasted through communicative validation in the discussion group. In addition, the researchers determined whether each subjective theory fosters or inhibits HCPB.

## 3 Results

Because subjective theories are commonly structured through intuitive explanatory and causal reasoning, several findings are presented as relationships perceived by participants between factors, behaviors, and consequences. In this sense, these statements should be interpreted as subjective explanations developed by the students.

### 3.1 Results of the communicative validation procedure

The students who took part in the discussion group confirmed the subjective theories found regarding HCPB and SEN. They shared experiences in which they engaged in HCPB toward peers with SEN, perceiving an increase in school participation, greater social acceptance, stronger socio-emotional skills, and increased psychological well-being for the beneficiaries. They stressed that defending and including peers with SEN entails a risk of bullying, however, they consider that it is fundamental to give that type of support to groups affected by high social vulnerability.

### 3.2 Results of the intra-case analysis

Table 2 summarizes the intra-case analysis. It presents a brief description of each case, its motto, and the main subjective theories (STs) found.

**Table 2:** Results of the intra-case analysis.

Case	Motto	Main STs: perceived causal beliefs
9th grade student, woman. Has a brother with SEN.	If you had a brother with SEN, you wouldn't like people to mistreat him.	1. HCPB entails including and protecting peers with SEN. 2. If the school teaches students about SEN, there will be more HCPB toward peers with SEN. 3. With HCPB, the beneficiary's self-esteem and academic performance improve.
9th grade student, woman. Had a classmate with SEN in primary school who was a target of exclusion.	If they had protected or included him, everything would have been better.	1. HCPB means defending and supporting peers with SEN. 2. When peer groups regard SEN as abnormal, you feel discouraged from defending and including peers with disabilities out of fear of violence and exclusion.
9th grade student, man. Has defended students with SEN. Has a family member diagnosed with autism.	It is bad for people to laugh at someone with SEN.	1. HCPB means protecting from negative remarks, defending, helping, and including a peer with SEN. 2. Since including and defending others gives you personal satisfaction, you focus on showing HCPB.
11th grade student, woman. Has a classmate diagnosed with autism.	If you are empathetic, you protect and accept a classmate with SEN.	1. HCPB consists in caring for, accepting, and supporting a student with SEN. 2. If there are opportunities for interaction with students with SEN, HCPB will occur. 3. HCPB could prevent the beneficiary from learning to defend themselves.
12th grade student, man, has a brother diagnosed with autism.	Helping those with SEN comes from your heart.	1. If your upbringing was good, you will engage in HCPB toward peers with SEN. 2. If you exhibit HCPB toward someone with SEN, they will be able to cope with their problems.
9th grade student, woman. Has defended students with SEN.	I think students with SEN can see me as a source of support.	1. Groups with positive norms promote HCPB. 2. HCPB has positive effects both for students with and without SEN, since defending and including others generates a safe environment.
9th grade student, woman. Has had classmates with SEN.	Including people is positive and appropriate.	1. Fear of physical violence inhibits HCPB. 2. HCPB could have an impact on students with SEN, leading them to become dependent as a result of overprotectiveness.
11th grade student, woman. Friend of a student with SEN who has been bullied.	In the end, they are people, and their disability should not be an issue.	1. HCPB means helping, protecting, and including students with SEN. 2. Empathetic upbringing and inclusion talks at school encourage us to defend peers with SEN.
12th grade student, woman. Defended a student with SEN, with whom she is now friends.	To feel supported is to feel safe.	1. HCPB means protecting and helping students with SEN during the hardest times. 2. Educational programs such as the School Integration Project and empathetic upbringing foster HCPB.
Focus group	To include is to live.	1. HCPB means protecting and including peers with SEN. 2. If a student with SEN betrays their benefactor, HCPB is inhibited. 3. HCPB creates a positive and safe environment for all students.

The statements represent the students' subjective theories, that is, their personal interpretations and perceived causal relationships.

### 3.3 Results of inter-case analysis

#### 3.3.1 Meaning high cost prosocial behavior toward students with SEN

Four subjective theories were found which convey the meaning of HCPB within the context of SEN. The first subjective theory defines this type of prosociality as a set of actions aimed at protecting and including students with SEN, who, apart from their learning problems, are affected by a high level of social vulnerability. The participants perceive that these actions entail social and personal risks, because they generate discrepancies, conflicts, and social disapproval among their peers. In addition, another subjective theory specifies that this helping behavior is characterized by an active, sustained disposition aimed at assisting peers with SEN when they are mocked and socially excluded.

The third subjective theory indicates that HCPB must also include academic support for those with SEN. This is exemplified by the inclusion of a classmate with SEN in group projects, with the participants helping them to understand the academic tasks or material involved. Students perceive that this type of help has high temporal, cognitive, social, and emotional costs, because the benefactor must devote additional time to give academic support to a peer with SEN, develop tolerance and patience, build capabilities to adapt the academic support provided, and deal with disapproval from other classmates. Interviewee 7 explained the situation as follows: “Yes, I did that. With that autistic classmate. Nobody wanted them in their group. With my friend, we included him, we did most academic stuff with him, I like him”.

Finally, the fourth subjective theory concerns values. The participants perceive that defending and including their peers with SEN essentially consists in pro-inclusion behavior, based on a commitment to equity and social justice in the educational system. With respect to this view, interviewee 3 noted: “It is important to include them, because that person finds it hard to act socially around other people, and it must be very tough for them and they need help, they need others to be there for them”. Table 3 describes the types of HCPB toward peers with SEN identified in the study.

**Table 3:** Types of HCPB.

Type of HCPB	Description
Protecting from physical violence	Directly intervening to stop blows or assaults
Defending from mockery	Intervening when peers instrumentalize a student with SEN to mock them
Confronting to stop verbal violence	Complaining and demanding an attacker to stop their harmful behavior
Informing school authorities to stop violence and/or exclusion	Turning to school authorities to stop the violence and/or exclusion
Providing academic support	Integrating in academic tasks, patiently explaining, reinforcing, and teaching
Advising	Orienting and guiding to prevent violence and exclusion
Offering friendship	Establishing bonds of friendship, even when risking exclusion and violence

With respect to the characteristics of the students who engage in HCPB, two subjective theories were found according to which those who exercise this type of prosociality are highly valued. Specifically, the students who defend and integrate peers with SEN are perceived as upright and kind people. Interviewee 4 expressed this view as follows: “It makes you a better person, they're kind-hearted and you can trust them”. Another subjective theory indicates that the students characterized high-cost prosocial behavior toward peers with SEN by attributing heroic traits to those who engage in it. Those who defend and integrate their peers with SEN are perceived as exceptional, empathetic, and righteous people who are willing to endure personal sacrifices. In this regard, interviewee 8 stated: “I mean, they are brave, because not just anybody is willing to do that”.

#### 3.3.2 Axial coding of the meaning HCPB

**Figure 1:**
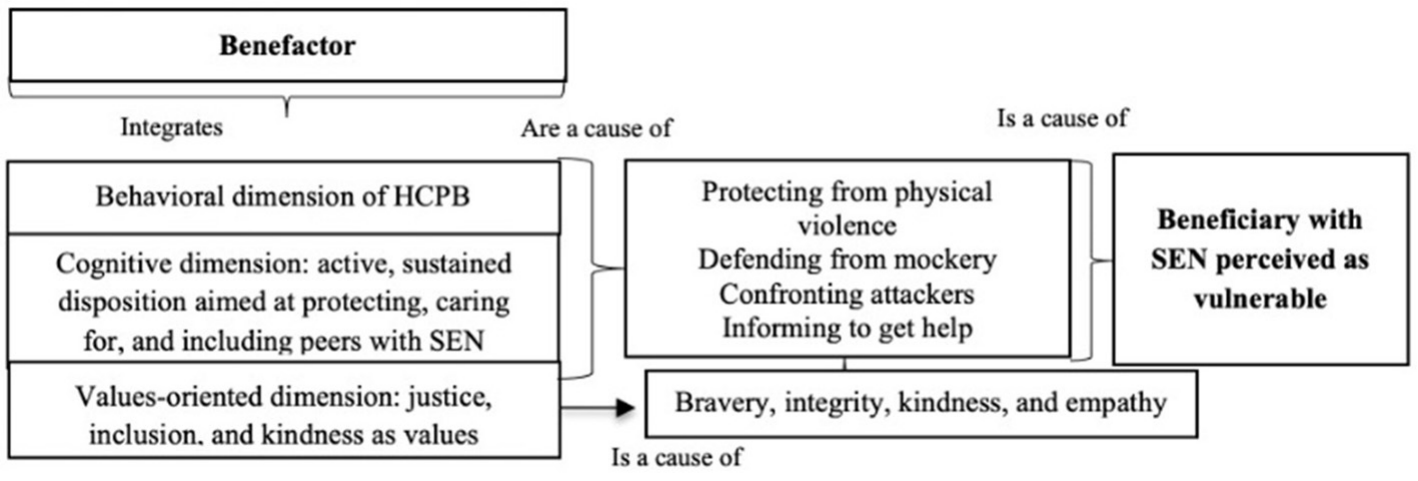
Meaning of HCPB toward students with SEN.

Based on the subjective theories identified, HCPB among secondary school students toward peers with SEN can be understood—according to participants' perceptions—across three dimensions. First of all, HCPB rests on an ethical dimension composed of several values: social justice, inclusion, and the pursuit of a kinder society. The cognitive dimension defines HCPB as involving risks, sacrifices, and persistence, as rendering help under these conditions requires systematic support that is not always understood by peers. Lastly, the behavioral dimension brings together multiple types of help that, when implemented, benefit the student with SEN (Figure 1).

#### 3.3.3 Subjective theories that promote or inhibit HCPB toward students with SEN

More subjective theories that identify factors that promote HCPB were found than theories linked to inhibitors. These subjective theories identify social, family, psychological, and school conditions associated with the deployment of costly prosociality within the context of SEN. For example, interviewee 7 explained that the risk of physical violence when defending a peer with SEN discourages this behavior: “It depends on the situation: just imagine, if I went there and they‘re beating somebody up, they'll beat me up too for trying to help”. In contrast, interviewee 5 explained that having peers who model this behavior provides safety and encourages one to defend and/or include a peer with SEN: “Seeing people who help motivates those who don't want to help…”. Table 4 describes the subjective theories presented in this section.

**Table 4:** Subjective theories that inhibit or promote HCPB.

Influencing factors	Subjective Theory (ST)	ST Description
Inhibiting factors		
Social factor	Participants perceived that defending and/or including peers with SEN, may lead to becoming a victim of school violence.	A social group with negative norms may respond violently when one defends or includes a peer with SEN
	Participants perceived that defending and/or including peers with SEN may lead to rejection or social exclusion.	A social group with negative norms may respond by rejecting or socially excluding the benefactor
Psychological factor	Participants perceived that if the beneficiary with SEN does not respond positively to help, the benefactor may experience negative emotions, which can inhibit HCPB.	When the beneficiary with SEN displays ingratitude and rejects the help provided, the benefactor experiences insecurity and shame.
Promoting factors		
Social factor	Participants perceived that when students model HCPB toward peers with SEN, others feel motivated to imitate this behavior.	Having peers who model HCPB eases the uncertainty surrounding the outcome of this behavior and increases confidence
	Participants perceived that when there are groups of students with positive norms, HCPB toward peers with SEN is valued and accepted.	If HCPB is valued, respected, and accepted by peers, costly help toward those with SEN is promoted
Psychological factor	Participants perceived that having personal or biographical experience with SEN fosters empathy and promotes HCPB.	This involves having biographical experience with SEN; for instance, having been classed as a student with SEN, having a family member with this status, or being friends with peers with SEN.
	Participants perceived that defending and including peers with SEN generates psychological well-being and self-efficacy, which in turn promotes HCPB.	HCPB produces relatively stable positive emotions in the benefactor –eudaimonic well-being– and trust in their capabilities
Family factor	Participants perceived that when families educate students about values such as inclusion, respect, and tolerance, HCPB toward students with SEN is more likely to occur.	Upbringing plays a key role in students' acquisition of values like friendship, team spirit, social justice, and inclusion
School factor	Participants perceived that when schools educate students about SEN, HCPB toward students with SEN is more likely to occur.	When the school educates students about SEN and encourages them to help peers with these issues, HCPB is intense and frequent

#### 3.3.4 Axial coding factors that inhibit or promote HCPB

**Figure 2:**
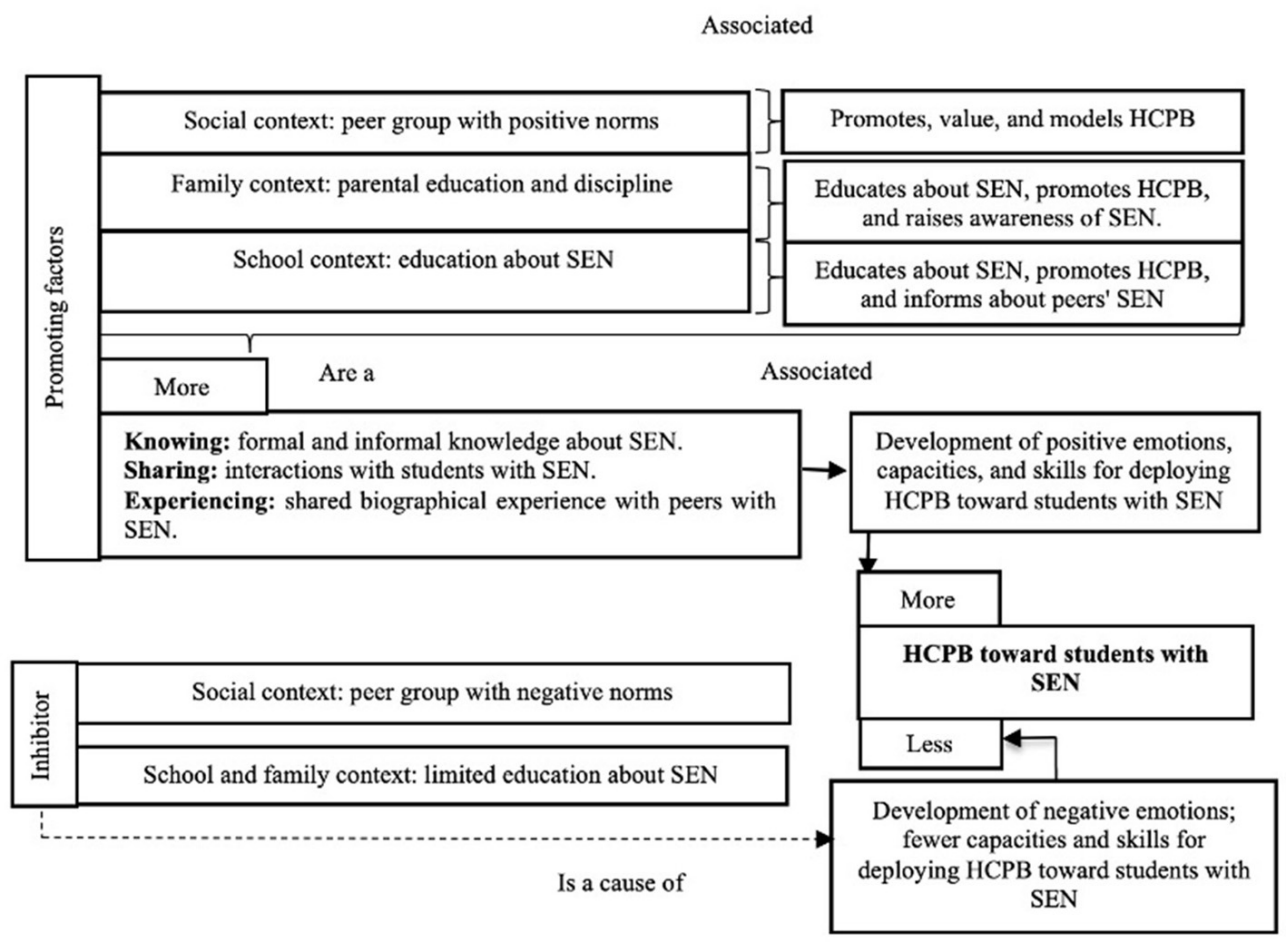
Factors that inhibit or promote HCPB toward students with SEN.

The subjective theories found indicate that knowing, spending time with, and having biographical experience with peers with SEN were described by participants as important conditions associated with engagement in HCPB. According to participants' perceptions, these conditions depend on the presence of groups (educators, peers, and family members) with positive norms, which value, educate about, and model HCPB toward people with SEN. When these conditions are met, participants described students as developing positive emotions associated with HCPB, in addition to capacities and skills that equip them to deploy this behavior. In contrast, the absence of these conditions inhibits HCPB toward students with SEN (Figure 2).

#### 3.3.5 Impact of HCPB according to students' subjective theories

The study revealed that students perceive an impact of HCPB on the benefactor, the beneficiary with SEN, and school climate. According to the participants in the study, for the student with SEN, being defended and/or included by their peers has a relevant impact on their social, educational, and psychological development. For instance, interviewee 3 explained: “She had an attention deficit and spent recess alone, and she had these crises… nobody helped her. One time, our peer group invited her to sit with us, she started spending recess with us, and she changed, the crises became less frequent, she started concentrating in class and all that.”

Regarding benefactors, participants in this study perceive some tensions, since HCPB toward peers with SEN has both positive and negative consequences. According to the interviewees, HCPB produces eudaimonic psychological well-being, that is, a lasting state of satisfaction when they defend and/or include students with SEN, together with a positive self-image. However, students who behave in this manner risk experiencing exclusion and violence, which leads to a reduction in hedonic psychological well-being and generates intense negative emotions when engaging in HCPB. Regarding this situation, interviewee 3 reported: “it felt nice, the teachers said 'she's changed thanks to you all' so we felt happier too”. Nevertheless, interviewee 8 also warned of a negative impact: “because we're often scared that they're going to pick on us, or say something hurtful, or hit us or something, for getting involved”.

Finally, students perceive that HCPB has a multidimensional positive impact on school climate, since it promotes students' acquisition of inclusive values, a pro-inclusion school culture, and positive interactions among peers. On this subject, interviewee 1 explained that, by defending and including their peers with SEN, “school climate will become more positive, because students will start reflecting on their actions”. Table 5 details the subjective theories identified within this thematic category.

**Table 5:** Perceived impact of HCPB according to students' subjective theories.

Argumentative structure of the ST	
Perceived cause	Perceived positive consequences for the beneficiary with SEN
Students perceived that when a student with SEN is supported through HCPB, the following may occur…	a) Emotional dimension and mental health: increased self-esteem, psychological well-being, and feelings of safety.
	b) Academic dimension: motivation to study, increased academic performance, participation in class, and engagement.
	c) Social dimension: social inclusion, school participation, and social recognition.
	Perceived negative consequences for the beneficiary with SEN
Students perceived that when a student with SEN is not supported through HCPB, the following may occur…	a) Emotional dimension and mental health: withdrawal, low-self-esteem, emotional crises, and self-destructive thoughts.
	b) Academic dimension: lack of academic motivation.
	c) Social dimension: social exclusion, inhibition of help-seeking behavior, and defensive-aggressive style.
	Perceived positive consequences for the benefactor
Students perceived that when a student defends and includes peers with SEN, the following may occur…	a) Psychological dimension: development of a positive self-image, trust in one's capabilities, bravery, moral integrity, and psychological well-being.
	b) Social dimension: acknowledgment and social recognition from school authorities (teachers, Deans of Discipline).
	Perceived negative consequences for the benefactor
	a) Psychological dimension: negative emotions, insecurity.
	b) Social dimension: mockery, humiliations, exclusion, and violence from peers.
	Perceived positive consequences for school climate
Students perceived that at school, when peers defend and include students with SEN, the following may occur…	a) Personal dimension: meaningful learning associated with positive attitudes toward peers with SEN.
	b) Interactional contextual dimension: empathetic interactions, reduction in conflicts, deeper interactional bonds.
	c) Organizational dimension: a safer school, imbued with values of justice and social inclusion.

#### 3.3.6 Axial coding subjective theories about the impact of HCPBI

**Figure 3:**
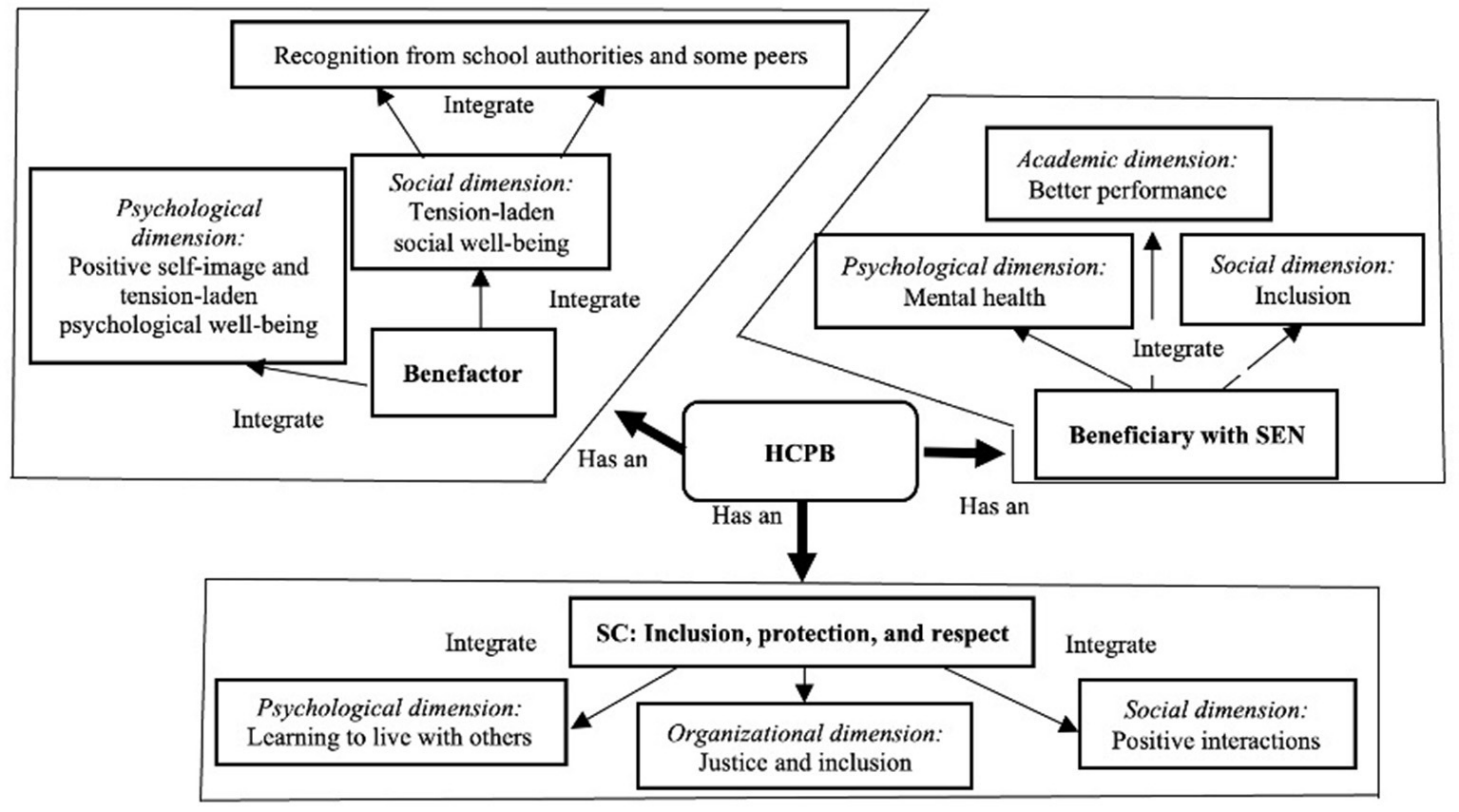
Axial coding of the impact of HCPB on school dynamics.

When a student engages in HCPB toward a peer with SEN, participants explained that there are consequences for the benefactor, the beneficiary, and school climate. According to the interviewees, the benefactor is perceived as experiencing an ambivalent effect. Basically, the psychological well-being produced by the helping behavior clashes with social well-being, since certain groups accept this type of behavior while others reject it. With respect to school climate and the beneficiary, students perceive that the impact is positive, fostering a culture of justice, inclusion, and permanent learning of ways to live with others, resulting in improved academic performance, psychological well-being, and inclusion for the beneficiary (Figure 3).

### 3.4 Selective coding of HCPB in secondary school students

**Figure 4:**
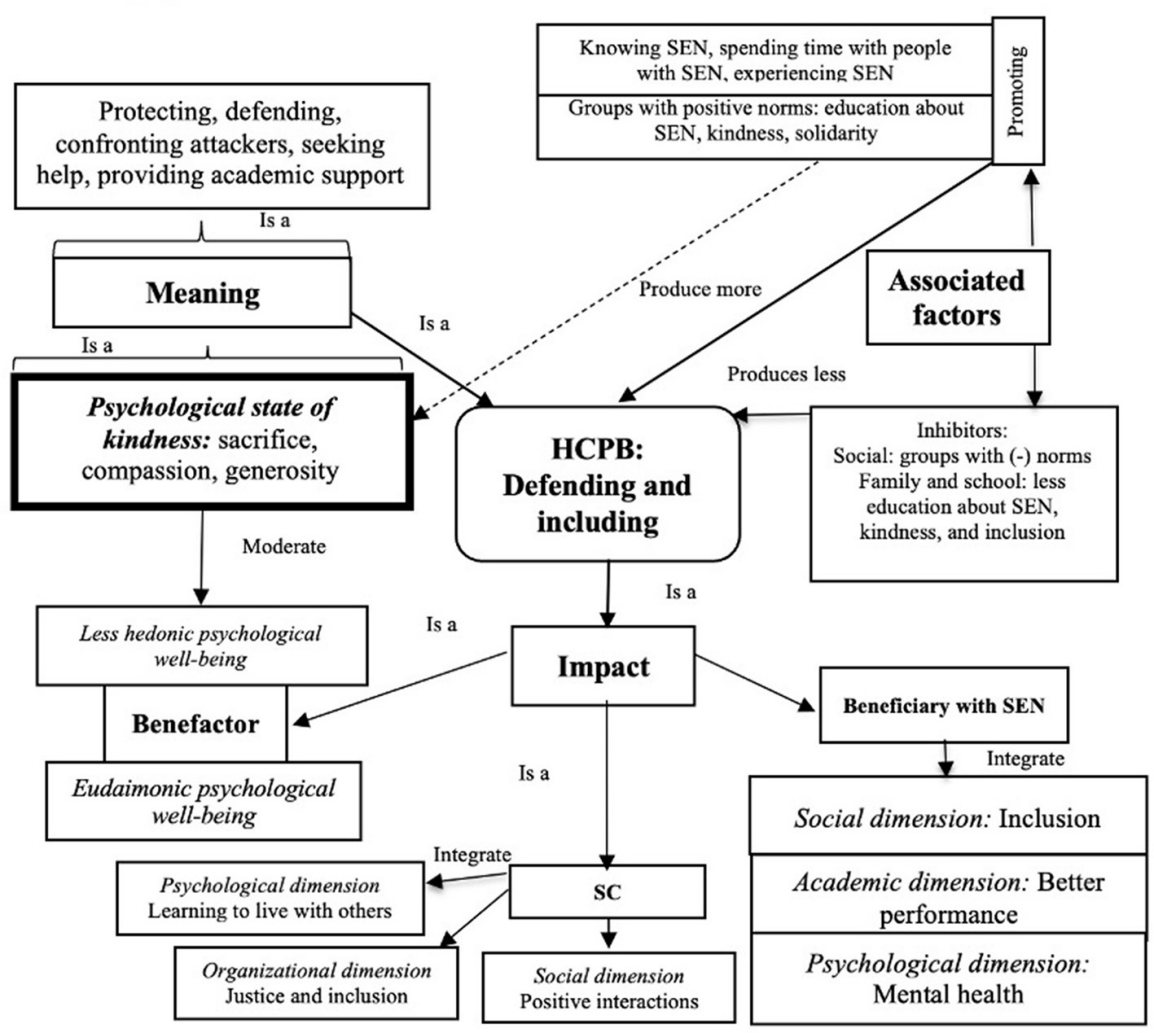
Selective coding of HCPB toward students with SEN.

Based on the participants of this study and applied to the context investigated, this model is organized around the central category defending and including, linked with a psychological state of kindness –a quality that humanizes the subject and prompts them to foster the other's well-being– that supports and orients this behavior. From the interviewees' perspective, factors such as contact with people with SEN and experiences of inclusion promote HCPB, whereas hostile social norms inhibit it. Students perceive that prosocial action has a dual effect on benefactors: a deterioration of hedonic well-being, sensitive to negative group dynamics, and the attainment of eudaimonic well-being, deeper and more stable, which reinforces their prosocial moral commitment. They also consider that HCPB generates a positive multidimensional impact on students with SEN and on school climate, boosting mutual care, justice, and inclusion (Figure 4).

## 4 Discussion

The general objective of this study was to describe and interpret the subjective theories that a group of students from a public secondary school in the Atacama Region, Chile, hold regarding HCPB toward students with SEN. Subjective theories were found which referred to the subjective meaning of HCPB that the students have constructed, arguments that justify this type of behavior toward peers with SEN, and explanations of its impact within the school context. Understanding how and why HCPB occurs in students who observe violence and social exclusion toward peers with SEN is crucial. Findings show that prosocial behavior has a positive impact on school climate (Wang et al., 2022), especially for students with SEN, who are markedly vulnerable to school violence (Carmona and Montanero, 2025).

The first specific objective of this study was to describe the subjective meaning of HCPB toward peers with SEN. The subjective theories indicate that this type of behavior consists in protecting and including students with SEN in social and academic activities, which, from the participants' perceptions in this study, entails a major personal toll for the benefactor associated with violence and discrimination risks. It was also found that this is a persistent behavior grounded in values such as social justice, kindness, and inclusion toward more vulnerable groups. (Padilla-Walker et al. 2018) and (Bergin et al. 2003) have distinguished this type of prosociality precisely due to the sacrifices and risks that the benefactor may experience, an observation echoed by the participants in this study. In addition, the reconstructed meaning provided by the interviewed students also indicates that the help rendered is persistent and that the benefactors are driven by pro-inclusion values, which can shed light on the dimensionality of this construct within the context of SEN.

Evidence shows that the cost of prosocial behavior has been linked to motivation to help others, with research showing that the costlier it is to help, the lower the motivation to engage in HCPB (Wiepking and Breeze, 2012). Thus, the high cost of defending and including peers with SEN that the participants report could explain the lower frequency of this type of behavior reported by (Nielson et al. 2017). Importantly, this finding shows that, from students' subjective theories, HCPB toward peers with SEN and the kindness-based inclination to help others are both driven by values. According to (Wu et al. 2021), this type of intrinsic motivation may be a central factor for the manifestation of this type of prosociality, and may also encourage others to join in. In line with the present study and according to students' subjective theories, this occurs because altruistic prosociality, genuinely motivated by the wish to benefit others, generates confidence and inspires the pursuit of the common good (Alvarado et al., 2019). Kindness, being a personal dimension of the subject, involves acting in accordance with a humanitarian moral framework, characterized by generosity, compassion, consideration for others, warmth, and friendliness (Bialobrzeska, 2024).

The axial model constructed makes it possible to understand the meaning that students attribute to HCPB considering its cognitive, behavioral, and values-oriented dimensions, the pillars of the types of HCPB expressed for the benefit of more vulnerable groups of peers. Interestingly, this model includes heroic qualities that were attributed by the students themselves to those who engage in HCPB and a set of values oriented by justice, kindness, and social inclusion. Drawing on prior literature, these dimensions are consistent with established conceptualizations of prosocial behavior; meanwhile, from students' subjective theories, additional meanings emerge that enrich this structure. Regarding the latter, (Siperstein et al. 2022), in a study on subjective theories for prosociality, also found that the values that underpin help-giving are essential for people to orient themselves prosocially. This model could explain the anti-violence attitude that (Willems et al. 2025) have found to be associated with prosociality, apart from suggesting how to develop an appropriate subjective meaning for encouraging students to engage in HCPB toward peers with SEN. In this regard, it is worth noting that students must acknowledge the limitations of the help that they can provide and seek adaptive ways to defend and include especially vulnerable school peers (Dell'Anna et al., 2025). According to (Bruno et al. 2020), students can inadequately interpret the guidelines for rendering help and implement mistaken measures to defend victims, generating a controversial impact at school.

The second specific objective was to determine the psychosocial factors that inhibit and promote this behavior from the perspective of the participants' subjective theories. On this topic, the study revealed psychological, social, family, and school factors that the interviewed students associate with high-cost prosociality, attesting to the complexity of this construct and providing evidence for the multidimensional model of prosocial behavior proposed by (Carlo and Padilla-Walker 2020).

More subjective theories that identify factors that promote HCPB were found than theories linked to inhibitors. With respect to the latter, school violence, rejection, and social exclusion are arguments that students use to justify the choice to avoid defending and including peers with SEN. These results are in line with those reported by (Woodgate et al. 2019). The theory of stigma by association could explain why students who defend and include peers with SEN can become victims of mockery and violence. This theory suggests that people who have contact with socially stigmatized individuals lose social worth, which can happen, for instance, due to knowing more people with mental health issues or having close relationships with them (Felix, 2024). This can be particularly relevant in adolescence, a period during which peer influence plays a major role in social adjustment (Wulandari et al., 2025). The findings obtained, from students' perceptions, show that stigma by association, within the context of HCPB toward adolescent peers with SEN, is exacerbated to the point of generating what could be termed “violence by association”.

In addition, the participants also reported factors that drive them to defend and include their peers with SEN. According to the proposed axial model, from students' subjective theories, developing the courage required to protect and include people with SEN is fundamentally based on knowing them, spending time with them, and sharing experiences with these peers. However, these conditions must be fostered, fundamentally, in a protected school environment where prosocial values and inclusion are systematically taught to prevent stigma by association. From the existing literature, it is also essential to develop students‘ emotional regulation capabilities for them to engage in high-cost prosociality (Bergin et al., 2003) and provide guidance for families to build an approach to parental education informed by values of justice and social inclusion.

The third specific objective was to describe and interpret how HCPB impacts on the student with SEN (the beneficiary), school climate, and the benefactor. The study revealed that, from the interviewees' perceptions, HCPB has a positive impact on the student with SEN on a school, social, and psychological level, while also helping to improve school climate. On this subject, (Woodgate et al. 2019) reported results in line with those of this study: when students include their peers with SEN, students‘ sense of belonging increases and their prejudices against disabilities are lessened; also, a more positive school climate is fostered (Wang et al., 2022). In contrast, in our study when HCPB toward students with SEN is limited, they become withdrawn and their self-esteem, social engagement, and academic performance decrease, which is consistent with the results reported in prior research (Cerda-Etchepare et al., 2022; Iqbal et al., 2021).

Another finding we identified is that, for the benefactor engaging in HCPB has both negative and positive consequences. Being a costly and risky behavior (Nielson et al., 2017; Padilla-Walker et al., 2018), students perceived that those who engage in it may be affected by social exclusion and violence. (Ángel 2018) and (Morales 2024) have also provided evidence that students who witness violence, when intervening in bullying incidents, face the latent fear of becoming victims due to getting involved. Nevertheless, based on the axial model generated from students' subjective theories, it was interpreted that this negative effect is likely to be situational, and that the positive impact on personal well-being and satisfaction due to helping vulnerable peers is eudaimonic, that is, more stable and permanent. Programs aimed at developing HCPB in students must consider the ambivalent impact on those who engage in these types of behaviors at school. Although participants often valued HCPB positively, their accounts also revealed tensions and possible unintended effects. Helping behaviors may occur within unequal social contexts, may expose benefactors to violence or exclusion, and in some cases may foster dependency or overprotection if not guided by autonomy-promoting practices. These findings highlight that HCPB is embedded within complex relational and structural conditions in school contexts.

For the fourth objective, a comprehensive model of HCPB was produced, based on the subjective theories found. In this model, from the interviewees' subjective theories, a psychological state of kindness, which is associated with positive norms in group settings, is the foundation of HCPB toward peers with SEN. This finding warns of the importance of gaining a clearer understanding of what kindness is, how it develops, and what its psychosocial implications are. Unfortunately, limited progress has been made in this respect in the social sciences, with difficulties still hindering the delimitation of the construct and a dearth of instruments preventing measurements (Shillington et al., 2026).

One of the limitations of this study was that the sample only included students from a public school, leaving out those from State-subsidized private or fully private institutions; therefore, the results presented can only be said to apply to public education. In addition, the sample was small, which may limit the transferability of the findings to other educational contexts with similar characteristics. Despite being situated in a Chilean public secondary school, this study provides elements transferable to global debates on inclusive education, since it shows how high-cost prosocial behavior among peers emerges in contexts of educational inequality. The theoretical sampling and the diversity of cases included—comprising students with varying levels of contact with classmates, friends, and family members with SEN, as well as students with no reported direct links—allow for a broader and more nuanced understanding of high-cost prosocial behavior in these contexts. The findings presented in this article are consistent with international inclusion frameworks, highlighting the role of schools in the promotion of social justice, student agency, and equitable participation (UNESCO, 2025b).

In addition to the previously mentioned limitations, it is important to acknowledge that participants' responses may have been influenced by social desirability bias, particularly given the moral nature of the phenomenon under study. Finally, as in any qualitative interpretative study, the analysis is subject to the researchers' interpretative decisions in the coding and reconstruction of subjective theories, which were addressed through team-based analysis and communicative validation to strengthen credibility.

In conclusion, based on the interviewees' subjective theories, HCPB entails significant sacrifices and costs for the students who defend and include peers with SEN. Nevertheless, it is fundamental to foster it systematically in schools, because it represents a contribution to school climate and to the psychosocial development of those who engage in this type of behavior. To promote HCPB in schools, it is necessary to consider the development of students‘ socioemotional skills and capacities as a way of mitigating the costs associated with help-giving.

## Data Availability

The raw data supporting the conclusions of this article will be made available by the authors, without undue reservation.

## References

[B1] Alvarado R. Pradenas C. Yáñez N. Cuadra D. Sandoval J. (2019). Teorías subjetivas del comportamiento prosocial: significados, desarrollo y motivaciones de jóvenes voluntarios ante un desastre socionatural. Liberabit 25, 251–266. Spanish. doi: 10.24265/liberabit.2019.v25n2.08

[B2] Álvarez-Guerrero G. García-Carrión R. Khalfaoui A. Santiago-Garabieta M. Flecha R. (2023). Preventing bullying of students with special educational needs through dialogic gatherings: a case study in elementary education. Humanit. Soc. Sci. Commun. 10, 1–9. doi: 10.1057/s41599-023-02470-8

[B3] Ángel N. G. (2018). Análisis bibliográfico de las características y consecuencias de los roles desempeñados en la violencia escolar: agresores, víctimas y observadores. Apuntes de Psicología 36, 181–190. Spanish. doi: 10.55414/jf65xp92

[B4] Arfah T. Tajuddin I. Ariani D. (2025). Why bystander act or do not act prosocially in bullying situations. Int. J. Educ. Adm. Manag. Leadersh. 5, 31–50. doi: 10.51629/ijeamal.v5i1.211

[B5] Arroyo G. Toro-Mayorga L. I. (2021). Interacción social entre los niños y niñas con necesidades educativas especiales y sus pares: una revisión narrativa. Revista Ecos de la Academia 7, 9–19. Spanish. doi: 10.53358/ecosacademia.v7i13.450

[B6] Bergin C. Talley S. Hamer L. (2003). Prosocial behaviors of young adolescents: a focus group study. J. Adolesc. 26, 13–32. doi: 10.1016/S0140-1971(02)00112-4 12550819

[B7] Bialobrzeska O. (2024). Niceness scale: development and validation of self-reported behavioral niceness measure. Pers. Individ. Dif. 218, 1–10. doi: 10.1016/j.paid.2023.112480

[B8] Bruno L. Joelsson T. Franzén A. Gottzén L. (2020). Heroes and others: tensions and challenges in implementing mentors in violence prevention in Swedish schools. J. Gend.-Based Violence 4, 141–155. doi: 10.1332/239868020X15881856376347

[B9] Carlo G. Knight G. P. McGinley M. Zamboanga B. L. Jarvis L.H. (2010). The multidimensionality of prosocial behaviors and evidence of measurement equivalence in Mexican American and European American early adolescents. J. Res. Adolesc. 20, 334–358. doi: 10.1111/j.1532-7795.2010.00637.x

[B10] Carlo G. Padilla-Walker L. (2020). Adolescents' prosocial behaviors through a multidimensional and multicultural lens. Child Dev. Perspect. 14, 265–272. doi: 10.1111/cdep.12391

[B11] Carmona Á. Montanero M. (2025). Bullying and social exclusion of students with special educational needs in primary education schools. Social Sciences 14, 1–18. doi: 10.3390/socsci14070430

[B12] Catalán J. (2018). Interpretación y cambio de teorí*as subjetivas*. La Serena: Universidad de La Serena.

[B13] Cerda-Etchepare G. Pérez-Wilson C. Serrano-Díaz N. Aragón-Mendizabal E. (2022). Necesidades educativas especiales en contextos vulnerables: incidencia de la convivencia escolar sobre el desempeño académico. Rev. Colomb. Educ. 86, 171–192. Spanish. doi: 10.17227/rce.num86-12450

[B14] Dell'Anna S. Entrich S. R. Banks J. (2025). Assessing the outcomes of students with special educational needs in inclusive education: a comparative study of Germany, Ireland, and Italy. Prospects 55, 1–15. doi: 10.1007/s11125-025-09730-2

[B15] Eisenberg N. Spinrad T. L. (2014). “Multidimensionality of prosocial behavior: rethinking the conceptualization and development of prosocial behavior,” in Prosocial Development: A Multidimensional Approach, ed. L. M. Padilla-Walker and G. Carlo (Oxford: Oxford University Press), 17–39.

[B16] Evans C. Smokowski P. (2015). Prosocial bystander behavior in bullying dynamics: assessing the impact of social capital. J. Youth Adolesc. 44, 2289–2307. doi: 10.1007/s10964-015-0338-5 26251101

[B17] Faúndez J. Figueroa I. Novoa C. Pérez J. (2025). Análisis de la matrí*cula escolar en Chile año 2025*. Santiago: Centro de Estudios Mineduc (CEM). Available online at: https://hdl.handle.net/20.500.12365/21779.~Spanish (Accessed November 10, 2025).

[B18] Felix E. (2024). Marked by association(s): a social network approach to investigating mental health-related associative stigma. J. Health Soc. Behav. 66, 259–275. doi: 10.1177/00221465241261711 39081161

[B19] Flick U. (2018). Introducción a la investigación cualitativa. Madrid: Ediciones Morata S.L. Spanish.

[B20] Gini G. Pozzoli T. Hauser M. (2011). Bullies have enhanced moral competence to judge relative to victims, but lack moral compassion. Pers. Individ. Dif. 50, 603–608. doi: 10.1016/j.paid.2010.12.002

[B21] Glaser B. Strauss A. (2006). The Discovery of Grounded Theory: Strategies for Qualitative Research. New Brunswick, NJ: Aldine Transaction.

[B22] Hammond S. Hill R. Edwards V. (2023). “Prosocial behavior in school contexts,” in The Cambridge Handbook of Prosociality: Development, Mechanisms, Promotion, ed. T. Malti and M. Davidov (Cambridge: Cambridge University Press), 442–458.

[B23] Hammond S. I. Brownell C. A. (2015). “Prosocial development across the lifespan,” in Encyclopedia on Early Childhood Development, ed. R. E. Tremblay, M. Boivin and R. D. Peters (Montreal: Abilio), 1–8. Available online at: https://www.child-encyclopedia.com/prosocial-behaviour/according-experts/prosocial-development-across-lifespan (Accessed November 3, 2025).

[B24] Holzer E. Moser E. (2025). The benefits of inclusive education: a systematic review of student achievement in secondary schools. Eur. J. Spec. Needs Educ. 1–19. doi: 10.1080/08856257.2025.2587615

[B25] Iqbal F. Senin M. Nordin M. Hasyim M. (2021). A qualitative study: impact of bullying on children with special needs. Linguist. Antverp. 12, 1443–1451.

[B26] Li B. Hu X. Chen L. Wu C. (2023). Longitudinal relations between school climate and prosocial behavior: the mediating role of gratitude. Psychol. Res. Behav. Manag. 16, 419–430. doi: 10.2147/PRBM.S395162 36819008 PMC9936877

[B27] Lin H.-M. Chu S.-Y. Chang W.-H. Lo I.-H. Peng H.-T. (2025). Promoting peer interaction and acceptance among students with special needs through an experiential learning program. Children 12, 1–14. doi: 10.3390/children12050543 PMC12109870 40426722

[B28] Lindstrom S. Waasdorp T. Debnam K. Bradshaw C. (2013). The role of bystander perceptions and school climate in influencing victims' responses to bullying: to retaliate or seek support? J. Criminol. 10, 1–10. doi: 10.1155/2013/780460

[B29] Milinga J. R. Possi M. K. (2015). Sighted students' prosocial behaviour towards assisting peers with visual impairment in Tanzania inclusive secondary schools. Int. J. Educ. Dev. 2, 15–40. doi: 10.25159/2312-3540/21

[B30] Morales J. A. (2024). Caracterización del perfil del victimario, la víctima y del observador en la trama de la violencia escolar. Rev. Cienc. Soc. 30, 516–533. Available online at: https://dialnet.unirioja.es/servlet/articulo?codigo=9603983.~Spanish (Accessed November 5, 2025).

[B31] Nielson M. Padilla-Walker L. Holmes E. (2017). How do men and women help? Validation of a multidimensional measure of prosocial behavior. J. Adolesc. 56, 91–106. doi: 10.1016/j.adolescence.2017.02.006 28192755

[B32] Nolen M. (2016). No good deed goes unpunished: the costs of helping others. [master's thesis]. Provo, UT: Brigham Young University.

[B33] Paciello M. Fida R. Cerniglia L. Tramontano C. Cole E. (2013). High cost helping scenario: the role of empathy, prosocial reasoning and moral disengagement on helping behavior. Pers. Individ. Dif. 55, 3–7. doi: 10.1016/j.paid.2012.11.004

[B34] Padilla-Walker L. M. Memmott-Elison M. K. Nielson M. G. (2018). Longitudinal change in high-cost prosocial behaviors of defending and including during the transition to adulthood. J. Youth Adolesc. 47, 1853–1865. doi: 10.1007/s10964-018-0875-9 29942985

[B35] Pfattheicher S. Nielsen Y. A. Thielmann I. (2022). Prosocial behavior and altruism: a review of concepts and definitions. Curr. Opin. Psychol. 44, 124–129. doi: 10.1016/j.copsyc.2021.08.021 34627110

[B36] Septarini B. G. Breen L. J. Hamamura T. (2025). Understanding prosocial behaviour: perspectives from different cultures and generations. Aust. J. Psychol. 77, 1–16. doi: 10.1080/00049530.2025.2507634 PMC12218546 40666226

[B37] Shillington K. Yates J. Johnson A. (2026). A scoping review of kindness measurement tools. Pers. Individ. Dif. 253, 1–9. doi: 10.1016/j.paid.2025.113616

[B38] Siperstein G. N. Ballard S. Jacobs H. Rodriquez J. Shriver T. (2022). A place for everybody: students' perspectives on inclusive behavior in school. Educ. Res. 51, 387–398. doi: 10.3102/0013189X221090509

[B39] Stake R. Visse M. (2026). Researching Care with Case Studies. London: Routledge.

[B40] Tomar A. (2023). Prosocial behavior and wellbeing among middle-aged individuals. Int. J. Novel Res. Dev. 8, 791–805. Available online at: https://ijnrd.org/papers/IJNRD2309093.pdf (Accessed November 20, 2025).

[B41] Ullah A. Samad A. (2025). The role of peer interaction in inclusive education for students with special needs. Ann. Methodol. Arch. Res. Rev. 3, 1–10. doi: 10.63075/tgjw7b62

[B42] UNESCO (2024). Save to learn and thrive: ending violence in and through education. United Nations Educational, Scientific and Cultural Organization. doi: 10.54675/LUPY3293

[B43] UNESCO (2025a). Qué debe saber acerca de la inclusión en la educación. Available online at: https://www.unesco.org/es/inclusion-education/need-know (Accessed March 02, 2026). Spanish.

[B44] UNESCO (2025b). Hacia una educación sin barreras: avances y desafí*os en la educación inclusiva que evidencian los resultados del SIRIED 2024*. Available online at: https://unesdoc.unesco.org/ark:/48223/pf0000396395 (Accessed March 02, 2026). Spanish.

[B45] UNICEF (2025). A Global Research Agenda for Children with Disabilities. Florence: Innocenti – Global Office of Research.

[B46] Waasdorp T. Fu R. Clary L. Bradshaw C. (2022). School climate and bullying bystander responses in middle and high school. J. Appl. Dev. Psychol. 80, 1–34. doi: 10.1016/j.appdev.2022.101412 PMC9015685 35444357

[B47] Wang C. Li B. Zhang L. Liu Y. Xu P. (2022). Prosocial behavior and teachers' attitudes towards bullying on peer victimization among middle school students: examining the cross-level moderating effect of classroom climate. Sch. Psychol. Rev. 53, 579–592. doi: 10.1080/2372966X.2021.2009313

[B48] Wiepking P. Breeze B. (2012). Feeling poor, acting stingy: the effect of money perceptions on charitable giving. Int. J. Nonprofit Volunt. Sect. Mark. 17, 13–24. doi: 10.1002/nvsm.415

[B49] Willems R. A. Sapouna M. De Amicis L. Völlink T. Dehue F. Dimakos L. . (2025). Encouraging positive bystander responses to bias-based bullying in primary schools through a serious game approach: a non-randomized controlled evaluation of the GATE-BULL program. Int. J. Bullying Prev. 7, 566–580. doi: 10.1007/s42380-024-00243-8

[B50] Woodgate R. Gonzalez M. Demczuk L. Snow W. Barriage S. Kirk S. (2019). How do peers promote social inclusion of children with disabilities? A mixed-methods systematic review. Disabil. Rehabil. 42, 1–28. doi: 10.1080/09638288.2018.1561955 30907279

[B51] Wu X. Wang X. Xu Q. Jin L. (2021). How the perceived cost of prosocial action inspires observers to contribute. Eur. J. Soc. Psychol. 52, 191–203. doi: 10.1002/ejsp.2824

[B52] Wulandari A. N. Ni'matuzahroh N. Istiqomah I. (2025). Self-esteem as a moderator of the influence of peer pressure on adolescent aggression. Psychol. Educ. Stud. 17, 53–63. doi: 10.17759/psyedu.2025170104

